# Mental health outcomes among early-entrance to college students: A cross-sectional study of an emerging educational system in the united states

**DOI:** 10.1192/j.eurpsy.2021.1070

**Published:** 2021-08-13

**Authors:** S. Singh, S. Singh, S. Narayanan, J. Kim, J. Jiao

**Affiliations:** 1 School Of Medicine, University of Missouri Kansas City, Kansas City, United States of America; 2 Outcomes Group, QUICK Research Institute, Kansas City, United States of America; 3 Department Of Psychiatry, Central Michigan University College of Medicine, Saginaw, United States of America; 4 College Of Life Sciences, University of California Los Angeles, Los Angeles, United States of America

**Keywords:** students, Anxiety, Depression, Education

## Abstract

**Introduction:**

In the United States, students who attend early-entrance to college programs (EECP) undergo a unique, accelerated educational path. Many of these programs require students to forego their final years of high school to take dual-enrollment classes while residing on a college campus. While previous literature has documented mental health outcomes among traditional college and high school student populations, there is scarce literature on the mental health among this hybrid population in the United States.

**Objectives:**

Investigate anxiety and depression among students enrolled in EECPs in the United States.

**Methods:**

Generalized Anxiety Disorder-7 item (GAD-7) and Patient Health Questionnaire-8 item (PHQ-8) were asked in 3 sets for how students felt before, during, and after their attendance in their EECP.

**Results:**

66 alumni students who graduated from an EECP were surveyed after giving informed consent. GAD-7 average scores before the students attended was 4.83 (median = 4, “mild anxiety”), during attendance was to 11.5 (median = 12, “moderately-severe anxiety”), and currently was 6.95 (median = 6, “moderate anxiety”). PHQ-8 scores for depression before attending were 5.1 (median = 4, “mild to potentially moderate depression”, during the program 10.9 (median 11.5, “moderately severe depression”), and current PHQ-8 was 16 (median = 16, “severe depression”).

**Conclusions:**

Anxiety and depression seem to have a presence in this student population, compared to traditional college student populations, but different compared to international cohorts. Academic rigor was a notable driving force of these outcomes, differing from the literature on traditional college student populations.

